# Spectrum of Presentations and Management Strategies in Renal Angiomyolipoma

**DOI:** 10.15586/jkcvhl.v9i1.221

**Published:** 2022-01-28

**Authors:** Sinan Khaddam, Shuchi Gulati

**Affiliations:** Division of Hematology and Oncology, Department of Medicine, University of Cincinnati, Cincinnati, OH, USA

**Keywords:** angiomyolipoma, epithelioid renal angiomyolipoma, mTOR inhibitors, PEComa

## Abstract

Renal angiomyolipoma (rAML) occurs rarely sporadically but is commonly encountered in patients with tuberous sclerosis complex and lymphangioleiomyomatosis. rAML is a rare entity, not seen regularly in daily practice; however, is commonly encountered and diagnosed by clinicians who approach and treat kidney masses. Basic knowledge of this entity is necessary to recognize that despite being benign, these tumors can rarely cause deadly complications such as hemorrhage or severe renal dysfunction or may have malignant components associated with them.

## Introduction

As the name implies, renal angiomyolipomas (rAMLs) are histologically derived from a variable number of vessels, spindle cells, and adipose tissue, and the nomenclature was first used historically by Grawitz in 1900 (1). Together with oncocytoma, rAMLs are the most common benign tumors in various surgical series (2). Eighty percent of rAMLs are sporadic without any genetic predisposition while the remaining ~20% coincide with the tuberous sclerosis complex (TSC) and lymphangioleiomatosis (LAM) (3). There is a lack of a strong level of evidence guiding treatment approaches for these tumors. The approach is primarily driven by clinical presentation and may contrast between active surveillance to invasive surgeries to systemic therapy if malignant. Diagnosis is easy on the latest imaging modalities available for renal masses, with the exception of low-fat rAMLs which are usually difficult to differentiate from renal cancers (4). WHO classifies rAML as belonging to the family of perivascular epithelioid cell neoplasms (PEComas) given their mesenchymal origin (5). These tumors share a molecular pathogenesis mechanism of inactivating *TSC1* or *TSC2* and other genes resulting in hyperactivation of the mammalian target of rapamycin (mTOR) pathway (6, 7). Here, we discuss the natural history, modes of presentation, and management strategies for rAML. We also underline the importance of recognizing the malignant potential of some rAMLs, especially the epithelioid variety as described in the case report in this issue of JKCVHL.

## Epidemiology, Pathogenesis, and Natural Course

In the era prior to the widespread availability of advanced imaging, the majority of rAMLs presented when they were large enough to cause symptoms (8). However, recently, due to access to imaging studies such as more rampant CT scans, the prevalence of rAML has increased. A study described 61,389 patients who underwent abdominal ultrasounds, where the overall prevalence or rAML was 0.44% (9). In general, the prevalence is higher in women and the tumors tend to occur at a young age, usually in the fourth or fifth decade (9–11). Young-onset rAMLs have however been reported in patients with TS and childhood cancer survivors (12). The natural history of rAML is controversial and varies between studies. The clinical course has been correlated with age at presentation, blood group, and location of tumor (13–15). The correlation between the size of tumors and growth is controversial. While some studies have reported larger tumors to grow faster (0.7 vs. 9.2 mm/year in one study), others have failed to identify this correlation (14, 16, 17). Two major histologic variants of rAMLs have been described: classic and epithelioid, and additionally a rare cystic variant (AML with epithelial cysts) (18–20). The epithelioid variant of rAML can rarely undergo malignant transformation and present with locally aggressive disease, including lymphadenopathy and distal metastases (3).

## Clinical Features of rAMLs

As described above, most rAMLs are incidentally diagnosed on scans obtained for other reasons and tend to be asymptomatic (11). Rarely, patients can present with abdominal pain, recurrent hematuria or severe manifestations such as renal insufficiency which are usually the scenarios when an intervention is required (1, 11). The scenario where rAMLs can become a medical emergency is the risk of life-threatening hemorrhage associated with the rupture of a large rAML mass. AMLs can present with spontaneous retroperitoneal hemorrhage (Wunderlich syndrome) (1). Bleeding risk is strongly associated with tumor vascularization and the presence of intra-tumoral aneurysms larger than 0.5 cm and requires immediate intervention (21).

## Diagnosis and Radiologic Classification

### Does every rAML mass need a biopsy?

The fat-containing feature of AMLs grants them a unique attenuation or echogenicity on imaging. For example, with rAMLs having multiple tissues interfaces and high vessel content, on ultrasound, they are almost always hyperechogenic (22). Thus, most AMLs can be diagnosed without a biopsy based on radiologic features, mainly fat content. As per the Song radiological classification, rAMLs can be divided into three types (fat-rich, fat-poor, and fat-invisible) (4). In fat-rich rAML, diagnosis can be established confidently without a need for further biopsy. About 5% of AMLs have very little fat content, imaging diagnosis becomes challenging and of a lower yield (23). These variants can appear like renal cell carcinoma (RCC) and a biopsy is usually warranted in these situations to distinguish the two. Some clinical and radiologic characteristics outside the kidney can be considered for differentiating clear cell RCC (ccRCC) from minimal fat rAML. For example, higher body mass index, older age, and male gender would more than likely dictate a diagnosis of ccRCC compared to rAML (24).

### Histopathology

After a biopsy, histology is not always straightforward in differentiating rAML from RCC. Epithelioid renal AML is rare and morphologically it is challenging to distinguish from ccRCC. Immunohistochemistry can help with distinction. Epithelial markers like cytokeratin are usually absent in rAMLs while they are more likely to be present in RCC. Smooth muscle markers such as vimentin and muscle-specific actin are commonly seen in rAMLs and melanocytic markers such as HMB-45 antigen and melan-A stains almost all AMLs (3).

### Prognostication of epithelioid rAML for malignant potential

The distinction of classic rAMLs from epithelioid AML (EAML) is essential because of the malignant potential of the latter entity. In a study, that examined 41 patients with pure EAML, metastasis was seen in ~50% of patients and 33% of patients died (25). However, due to the rarity of this entity, there is no consensus on pathognomonic clinical features, imaging characteristics, or histopathologic features to classify the malignant potential of EAMLs. A retrospective study compared clinical features of patients with classic rAML (n = 204) and EAML (n = 27) (26). Male sex, younger age at diagnosis, and large size of tumor predicted for a higher likelihood of the tumor being EAML. Radiologic examination (MRI) of AMLs from 12 patients, suggested the presence of an exophytic growth pattern, a solid lesion, hemorrhage, enlarged vessels, heterogeneous hyperintensity on diffusionweighted imaging, and rapid washout pattern to be associated with EAML (26). Other retrospective studies have attempted to delineate pathological features associated with malignant behavior. In a retrospective study of 41 cases from multiple institutions, a combination of clinical and pathological features to help classify EAML into low risk (<2 parameters), intermediate risk (2–3 parameters), and high risk (>3 parameters). These features included: tumor size >7 cm, extrarenal extension (or renal vein involvement), the concurrent presence of TS or presence of concomitant classic angiomyolipoma, necrosis, and carcinoma-like growth pattern (25). Another study that included 40 patients, described four features (≥70% atypical epithelioid cells, atypical mitotic figures, ≥2 mitotic figures per 10 high power field, necrosis) (27). The presence of more than three features was shown to be predictive of malignant behavior.

## Management of rAML: Different Scenarios and Latest Developments

### Sporadic rAML

The choice to switch from surveillance to start treatment depends on the presence of symptoms as well as tumor size (>4–6 cm is an accepted cut-off, although controversial) (13). Treatment options include surgery and embolization of blood vessels. A priority in the treatment of rAML is the preservation of kidney function (nephron sparing) by attempting less radical local treatments. A lot of minimally invasive procedures are proving to be effective in case reports and case series for AML treatments with examples including transarterial embolization and percutaneous cryoablation (28, 29). Acute life-threatening hemorrhage generally requires urgent selective renal artery embolization to reduce further hemorrhage, clinical stabilization, and eliminating the need for invasive therapy (30). Unlike hereditary TS-associated rAMLs, there is no data or evidence to support the use of systemic drugs such as mTOR inhibitors in sporadic rAMLs.

### Malignant PEComas

Malignant PEComas are rare, the estimated incidence is 0.12, 0.24/1,000,000 and should be managed in a center specialized to manage sarcomas (31). In a retrospective study, radical surgery with microscopically clear margins has been shown to be the only curative regimen for PEComas as they are known to be chemotherapy and radiation therapy-resistant (31, 32).

### mTOR inhibitors in PEComas

Older studies have established that mTOR pathway activation is common in AML and PEComas even if they are TS unrelated (33). Studies evaluating conventional chemotherapy and conventional targeted agents are limited. A retrospective study of advanced PEComas treated between 2000 and 2018 that included 53 patients in five centers in Europe described the efficacy of three different classes of drugs (34). The 53 cases described in this study included six patients with rAML, the remaining were retroperitoneal, uterine, gastrointestinal, soft-tissue, pelvis, and lung. Chemotherapy agents, gemcitabine and anthracycline regimens had modest and similar efficacy [overall response rate (ORR) with gemcitabine was 20% and progression-free survival (PFS) of 3.4 months vs. an ORR of 13% with an anthracycline with a similar PFS of 3.2 months]. Antiangiogenic tyrosine kinase inhibitors (pazopanib, sorafenib, and sunitinib) led to an ORR of 8.3%, PFS: 5.4 months. In comparison, mTOR inhibitors (everolimus, sirolimus, and temsirolimus) showed the highest efficacy with an ORR of 41% and PFS of 9 months. Eleven (28.2%) patients had a durable response that lasted more than 1 year in the mTOR inhibitor group, thus establishing the role of mTOR inhibitors in the front-line setting for recurrent, advanced, and metastatic PEComas. Chemotherapy (anthracycline and gemcitabine-based regimens) and antiangiogenic treatments would be reasonable options for subsequent lines of treatment. Another study analyzed 15 consecutive patients with PEComa, who received sirolimus up-front (11 patients) or received doxorubicin-based chemotherapy in the front-line and sirolimus in subsequent lines (four patients) (35). The ORR was 73% in the sirolimus arm, while only one patient had a response in the chemotherapy arm. Median PFS was not reached for sirolimus [was 4.9 months (95% CI: 3.8-NA) for first-line chemotherapy]. All patients treated with mTOR inhibitor achieved disease control. This study further supports the role of mTOR inhibitors in PEComas.

While oral treatments are tempting options, oral mTOR inhibitors treatment usually needs monitoring and might not achieve reliable absorption and sufficient intra-tumoral concentration. This has led to the evaluation of the novel agent nab-sirolimus which is an intravenous (IV) preparation in patients with malignant PEComas (36). Nab-sirolimus was studied in a phase-2 clinical trial (AMPECT) that included four patients with kidney PEComa. In the 34 evaluable patients, an ORR of 39% was reported, including response seen in three of the renal PEComa patients (36). Based on this trial, the FDA approved nab-sirolimus as the first drug specifically indicated for the treatment of patients with malignant PEComas.

### Immunotherapy in epitheliod rAML

Histologically, AMLs show consistent staining for HMB-45, an antibody usually used in immunohistochemistry diagnosis of malignant melanoma, a tumor with a high response rate to immunotherapy (37). With the rarity of malignant epithelioid rAML and malignant PEComas in general; barring case reports, there is scarce data to the best of our knowledge describing the role of immunotherapy in epithelioid AML (38, 39). These reports do show a durable response when nivolimab or pembrolizumab were used in subsequent lines of treatment for epithelioid rAML or malignant PEComa. These responses have happened in the context of the highest tumor PDL1 expression and brisk T cells infiltration. Further genomic analysis of epithelioid tumors and identification of biomarkers of response to immunotherapy-based drugs may pave the path for future clinical trials in these rare but malignant tumors.

### rAML in the setting of tuberous sclerosis

As previously described, the majority of AMLs are sporadic, up to 20% are associated with hereditary conditions such as TSC and LAM (8). Management of these lesions differs from that of the sporadic rAMLs as they present with multiple and bilateral tumors and seem to respond well to mTOR inhibitors. FDA approval for drugs to be used in the treatment of rAML in the context of tuberous sclerosis preceded the approvals in sporadic cases. A phase-3 clinical trial (EXIST-2) enrolled 118 patients who had at least one angiomyolipoma greater than 3 cm in size and a diagnosis of TS or sporadic LAM (40). Patients were randomized in a 2:1 manner to receive everolimus versus placebo. Ninety-two of the 118 patients had AMLs in both kidneys. Patients who received everolimus achieved an ORR of 42% compared to a 0% response rate in the placebo group with an acceptable safety profile (the most common adverse event noted was stomatitis). Everolimus received accelerated FDA approval for rAML associated with TS, not in need of immediate surgery (41). Efficacy of everolimus was further studied at a lower maintenance dose of 5 mg in a subsequent study (42). Patients in this study received the standard “induction” dose of 10 mg followed by staying on a “maintenance dose” of 5 mg. All 24 patients enrolled in this study showed a response with decreased tumor volume and >50% reduction of volume was seen in 12 out of 24 patients. The lower dose used here was associated with fewer side effects.

Based on these studies, for TS-associated rAMLs, everolimus use is recommended in the first-line prophylactic setting at a dose of 10 mg oral daily, proposed to be started at a tumor size cutoff of 3 cm (40). It is important to note that the use of mTOR inhibitors in TS-associated rAML does not need the tumors to be malignant (compared to approval for malignant PEComas).

### Hormonal manipulation in rAML

There are some case series that report an association between the use of hormonal therapy rAML growth (43). Some reports from the obstetrics literature have shown a tendency for rAML to rupture during pregnancy (44). There are clinical case reports describing the expression of estrogen and progesterone receptors on rAMLs, thus raising the possibility of clinical benefit from hormonal manipulation treatment, although this approach has not formally been investigated in rAML patients (45).

### Surveillance in rAML

Surveillance, in general, is recommended in rAML based on the variability of its clinical behavior as described in the previous sections. The frequency of surveillance differs depending on the size and the histologic variance. Ultrasound is preferred in low-risk and small-size AMLs and the surveillance frequency increases from every 3–4 years ultrasound for small tumors below 2 cm to every 6–12 months in larger tumors (46). After resection, tumors that show histology of epithelioid variant with high-risk features, whole body CT is recommended and possibly additional abdominal MRI 6 months post-surgery and then yearly for 5 years (17). In patients with synchronous bilateral rAMLs, referral for genetic testing and evaluation should also be discussed with patients, as ~20% of AMLs can be associated with TS or LAM-like conditions (13).

## Conclusions

Renal angiomyolipoma is a benign tumor but rarely can present with an aggressive malignant variant. It has a unique radiologic and histologic signature. Understanding the molecular pathology of this tumor uncovers the importance of mTOR pathway and has improved our utilization of targeting therapeutics in tumors of malignant potential and those that are associated with hereditary conditions such as tuberous sclerosis. mTOR inhibitors have gained approval and acceptance as a possible treatment modality for these tumors while there is a scarcity of data pertaining to immunotherapy, even though the available case reports are promising. There is still no consensus on how frequently to monitor patients with sporadic rAML. Further studies to characterize these tumors at a molecular level will lead to better insight into the pathogenesis and management of these rare but clinically important tumors.
